# Conventional Western Treatment Combined With Chinese Herbal Medicine Alleviates the Progressive Risk of Lung Cancer in Patients With Chronic Obstructive Pulmonary Disease: A Nationwide Retrospective Cohort Study

**DOI:** 10.3389/fphar.2019.00987

**Published:** 2019-09-13

**Authors:** Tsai-Hui Lin, Shu-I Chen, Yuan-Chih Su, Mei-Chen Lin, Hung-Jen Lin, Sheng-Teng Huang

**Affiliations:** ^1^Department of Chinese Medicine, China Medical University Hospital, Taichung, Taiwan; ^2^Management Office for Health Data, China Medical University Hospital, Taichung, Taiwan; ^3^School of Chinese Medicine, China Medical University, Taichung, Taiwan; ^4^An-Nan Hospital, China Medical University, Tainan, Taiwan; ^5^Chinese Medicine Research Center, China Medical University, Taichung, Taiwan; ^6^Research Center for Chinese Herbal Medicine, China Medical University, Taichung, Taiwan; ^7^Cancer Research Center for Traditional Chinese Medicine, Department of Medical Research, China Medical University Hospital, Taichung, Taiwan

**Keywords:** lung cancer, chronic obstructive pulmonary disease, Chinese herbal medicine, retrospective cohort study, traditional Chinese medicine

## Abstract

**Background and purpose:** Lung cancer has high global incidence and mortality rates. Chronic obstructive pulmonary disease (COPD) is strongly associated with lung cancer and is an independent risk factor for lung cancer with or without smoking. Chinese herbal medicines (CHMs) are used to treat COPD. This study sought to determine whether CHM treatment effectively decreases the incidence of lung cancer in COPD patients receiving conventional Western medical treatment.

**Methods:** Records obtained from the National Health Insurance Research Database (NHIRD) were used to identify 81,780 adults aged ≥18 years newly diagnosed with COPD in Taiwan between 2000 and 2010. Among them, 11,180 received CHMs after COPD diagnosis and 23,319 did not (non-CHM). After excluding patients with missing basic demographic information, each group consisted of 2,682 patients. Statistical methods analyzed the baseline characteristics for both groups and we performed a Cox proportional hazard regression analysis to examine the incidence of lung cancer. The cumulative incidence of lung cancer in COPD patients with or without CHM treatment was calculated by the Kaplan-Meier method. The association between herbs and formulas was examined by NodeXL to perform a network analysis of CHM.

**Results:** COPD patients using CHM had a lower risk for lung cancer (adjusted hazards ratio [aHR] = 0.36, 95% confidence interval [CI] = 0.24–0.53, p < 0.001). Older age was associated with a higher risk of lung cancer: patients aged 40–59 years (aHR = 5.32, 95% CI = 2.19–12.94, p < 0.001) and those aged ≥60 years (aHR = 16.75, 95% CI = 7.54–37.23, p < 0.001) were at significantly greater risk compared with patients aged 18–39 years. CHM use was associated with a trend for a lower cumulative incidence of lung cancer compared with non-CHM use (p < 0.001). Among the 10 most commonly used single herbs and formulas used to decrease the risk of lung cancer in COPD patients, *Fritillariae thunbergii* was the most commonly used single herb and Xiao Qing Long Tang the most commonly used formula.

**Conclusion:** The findings from this nationwide retrospective cohort study indicate that CHM as adjunctive therapy in COPD treatment regimens may reduce the risk of lung cancer in this vulnerable patient population.

## Introduction

An estimated 2.1 million lung cancer diagnoses and 1.8 million deaths worldwide were attributed to lung cancer in 2018, accounting for around 11.6% of the total cancer burden, making it the most common cause of cancer-related deaths (Bray et al., 2018). According to American Cancer Society statistics for 2017, the numbers of deaths due to lung and bronchus cancers in the United States were estimated to account for around one-quarter of all cancer deaths in 2017 (27% among men and 25% among women) (Siegel et al., 2017). The five-year survival rate correlated with disease stage at diagnosis, with higher rates of five-year survival for patients with early-stage disease compared with those diagnosed with late-stage disease. Although low-dose computed tomography screening for lung cancer can reduce lung cancer mortality rates by up to 20% as a result of early diagnosis and subsequent early treatment (Aberle et al., 2011), the five-year survival rate averages only 18% overall, regardless of disease stages (Siegel et al., 2017).

Chronic obstructive pulmonary disease (COPD) is recognized as being an independent risk factor for lung cancer, with or without smoking (Takiguchi et al., 2014). A reduction in averaged forced expiratory volume in one second (FEV_1_) values is associated with increased risks of lung cancer and mortality. Known risk factors that are highly correlated with risk of COPD and lung cancer include cigarette smoking, old age, and familial susceptibility. A review of related mechanisms underlying the disease processes in COPD and lung cancer found that each cigarette puff contains approximately 10^15^ free radicals, including reactive nitrogen and oxygen species (RNOS), which damages DNA and leads to inflammation in multiple pathways (Durham and Adcock, 2015). Such inflammation may persist for decades after smoking cessation; aberrant inflammation and immunity has been found to gradually lead to the development of lung cancer (Adcock et al., 2011; Bozinovski et al., 2016). Moreover, telomere shortening has been found in both COPD and lung cancer and short telomere length may undermine the tumor-suppressing mechanism and contribute to inflammatory reaction. COPD is caused by chronic inflammatory response, matrix degradation, apoptosis, remodeling in the airways, and lung parenchyma. Increasing evidence shows that COPD may be a driver of lung cancer. Possible mechanisms involve mitochondrial dysfunction affecting endothelial cell apoptosis, inflammatory mediators influencing the tumor microenvironment, sustained activation of the NF-κB pathway, repeated damage and repair processes that increase the likelihood of EMT and hypoxic status inducing the activation of the alpha subunit of the transcription factor hypoxia-inducible factor-1 (HIF-1α), which is positively correlated with the severity of airflow limitation in COPD and associated with tumor metastasis, angiogenesis, and poor prognosis (Tao et al., 2014; Durham and Adcock, 2015; Masoud and Li, 2015; Bozinovski et al., 2016).

Traditional Chinese medicine (TCM) has been used for centuries to treat the symptoms and signs of COPD, with benefits including a better quality of life, improvement in lung function and a lower risk of exacerbation, and a higher exercise capacity, compared with no TCM use (An et al., 2012; Haifeng et al., 2015). Chinese herbal medicine (CHM) is an important category of TCM. A meta-analysis of evidence on the anti-inflammatory impact of CHM in patients with COPD has shown that CHM treatment leads to reductions in serum levels of interleukin (IL)-6, IL-8, tumor necrosis factor alpha (TNF-α) and transforming growth factor beta 1 (TGF-β1), as well as decreases in sputum levels of IL-8 and TNF-α (Miao et al., 2016). In a cigarette smoke-induced mouse model of COPD, the CHM formula “Liujunzi Tang” indicated that it possesses anti-inflammatory and anti-oxidative properties through its inhibition of NF-κB activation and subsequent protection of lung function (Zhou et al., 2016). A water-soluble derivative of tanshinone IIA (Tan IIA) appears to decrease levels of cigarette smoke-induced inflammation and oxidative stress by inhibiting the HIF-1α/MAPK signaling pathway (Guan et al., 2018). In a randomized controlled trial involving older patients with COPD and FEV_1_/forced vital capacity (FEV) values of less than 70%, significant reductions in serum CRP were observed in the Hochuekkito “Bu-Zhong-Yi-Qi-Tang” treatment group, leading to improvements in systemic inflammation (Shinozuka et al., 2007). However, no evidence as yet has reported that CHM treatment in COPD reduces the incidence of lung cancer.

Since its inception in 1995, Taiwan’s National Health Insurance (NHI) program has integrated CHM into mainstream medicine. The purpose of this study is to investigate whether the use of CHM in patients with COPD could help to reduce the risk of lung cancer. The study also sought to determine which formulation(s) of CHM appear to be most useful, for future investigations seeking to develop more effective treatments.

## Materials and Methods

### Study Design and Data Source

We obtained anonymized, de-identified patient data from the National Health Insurance Research Database (NHIRD) for this retrospective cohort study. The NHIRD has recorded health insurance information from Taiwan’s NHI program since 1996. The NHI covers medical procedures such as drugs and surgeries in clinical visit or hospitalizations. TCM outpatient use is also included in the NHI program. This study was approved by the Review Board and Ethics Committee of China Medical University Hospital, Taichung, Taiwan (CMUH104-REC2-115(CR-2)). The review board waived the requirement to sign informed consent from patients, as all of the data were de-identified.

The main data source consists of the Longitudinal Health Insurance Database 2000 (LHID2000), which contains medical care data for a cohort of 1 million insured individuals from 1996 through 2013. Details recorded in the database include the International Classification of Diseases, 9^th^ revision, Clinical Modification (ICD-9-CM) diagnosis codes, details of drug prescriptions and surgery, gender and age.

### Subjects

The study population consisted of patients with COPD (ICD-9-CM codes: 491, 492, 494, and 496) between the years 2000 and 2010, classified into two cohorts according to whether or not they received CHM after a new diagnosis of COPD between 2000 and 2012; Group 1: CHM users, consisting of TCM outpatients with at least 14 days of CHM prescriptions; Group 2: COPD patients with no record of any TCM clinical visits. The study population excluded individuals with any missing basic demographic information or who were aged less than 18 years.

### Primary Outcome, Comorbidities, and Demographic Covariates

The primary outcome of this study was a new diagnosis of lung cancer (ICD-9-CM: 162) after COPD. Any life-threatening illnesses such as malignant neoplasms were verified by pathological data. We evaluated the incidence of the following comorbidities at baseline: hypertension (ICD-9-CM: 401-405), stroke (ICD-9-CM: 430-438), coronary artery disease (ICD-9-CM: 410-414), diabetes mellitus (ICD-9-CM: 250), chronic kidney disease (ICD-9-CM: 585), osteoporosis (ICD-9-CM: 733), depression (ICD-9-CM: 296.2, 296.3, 296.82, 300.4, 309.1, 309.28, 311), anxiety (ICD-9-CM: 300), hyperlipidemia (ICD-9-CM: 272), smoking-related diseases (ICD-9-CM: V15.82, 305.1, 794.2, 518.1, 518.3, 518.4) and dementia (ICD-9-CM: 290, 294.1, 331.0), for risk-adjusted outcome analyses. Demographic covariates that were assessed at baseline included gender, age, and work occupations (**Supplementary Table 1**).

### Propensity Score Matching

Patients were divided into two groups: those who were prescribed CHM and those who were not. A propensity score was generated for each patient based on gender, age, work occupation and comorbidities, and logistic regression analysis examined the likelihood of lung cancer in the two matched groups.

### Statistical Analyses

Baseline characteristics between the CHM and non-CHM cohorts were compared using a Student’s *t*-test for continuous variables and a Chi-square test for categorical variables. Cox proportional hazard regression analysis was performed to test for the occurrence of lung cancer. The 10 most commonly prescribed herbs and formulas for treating COPD were selected according to total person-days. We calculated the cumulative incidence rates of lung cancer for the CHM and non-CHM groups using Kaplan-Meier analysis. Network plot demonstrated the usage patterns of each two CHMs, using open-source freeware NodeXL (http://nodexl.codeplex.com/). The statistical analysis was run with type I error α = 0.05, using the statistical software package SAS version 9.4 (SAS Institute Inc., Cary, NC, USA).

## Results

### Demographic Characteristics

**Figure 1:**
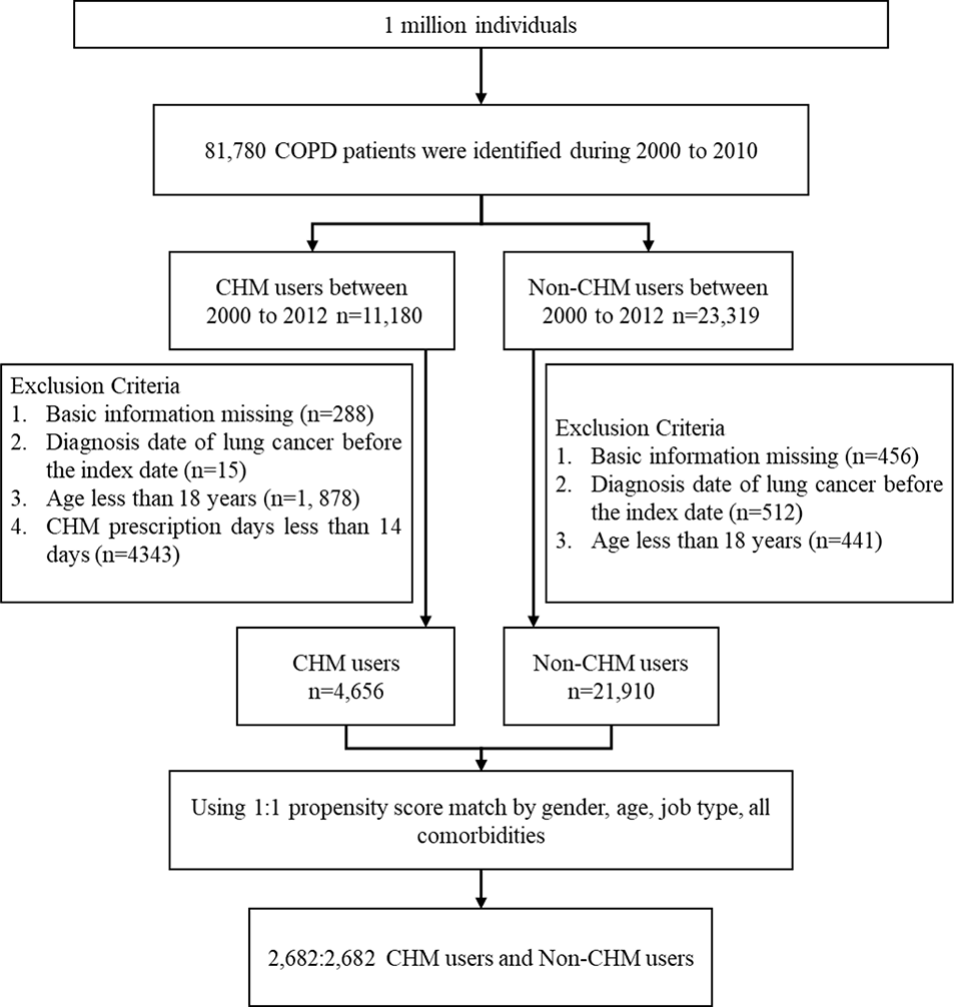
Flow chart of study cases identified from 1 million insured individuals in Taiwan between 2000 and 2010.

Of the 81,780 patients with COPD identified in the NHIRD, 11,180 were CHM users and 23,319 were non-CHM users between 2000 and 2010 (**Figure 1**). Fifteen patients in the CHM cohort and 512 in the non-CHM cohort were identified as having lung cancer before the index date and were excluded from analyses. After applying propensity score-matched (1:1) analysis, the final study population consisted of 2,682 CHM users and 2,682 non-CHM users. **Table 1** details basic demographic details for each group. In each group, over half of the participants were male (56.1% in the CHM group; 54.9% in the non-CHM group). Compared to non-CHM users, CHM users were older (mean, 57.7 years in the CHM cohort vs 55.9 years in the non-CHM cohort, p < 0.001) and more likely to have hypertension (p = 0.03).

**Table 1:** Demographic characteristics and comorbidities of patients newly diagnosed with COPD in Taiwan during 2000–2010.

	Non- CHM		CHM		p value
Variable	N = 2682		N = 2682		
	50.00%		50.00%		
	n	%	n	%	
**Sex**					0.39
Female	1,209	45.1	1,178	43.9	
Male	1,473	54.9	1,504	56.1	
Age at baseline					<0.001
18–39	561	20.9	336	12.5	
40–59	991	37.0	1,084	40.4	
≥60	1,130	42.1	1,262	47.1	
Mean(SD)	55.9	17.9	57.7	14.7	<0.001
Job type					0.68
Office workers	1,299	48.4	1,267	47.2	
Manual workers	1,124	41.9	1,149	42.8	
Others	259	9.7	266	9.9	
Baseline comorbidity					
Hypertension	1,092	40.7	1,170	43.6	0.03
Stroke	178	6.6	186	6.9	0.66
Coronary artery disease	624	23.3	676	25.2	0.10
Hyperlipidemia	735	27.4	746	27.8	0.74
Diabetes mellitus	259	9.7	272	10.1	0.55
Chronic kidney disease	60	2.2	58	2.2	0.85
Osteoporosis	312	11.6	354	13.2	0.08
Depression	168	6.3	194	7.2	0.16
Anxiety	568	21.2	605	22.6	0.22
Dementia	45	1.7	39	1.5	0.51
Smoking related diseases	11	0.4	13	0.5	0.68

Chi-square test, ‡Student’s t test.

CHM, Chinese herbal medicine; SD, standard deviation.

### Cause-Specific Hazard Ratios and 10-Year Cumulative Incidence of Lung Cancer

**Figure 2:**
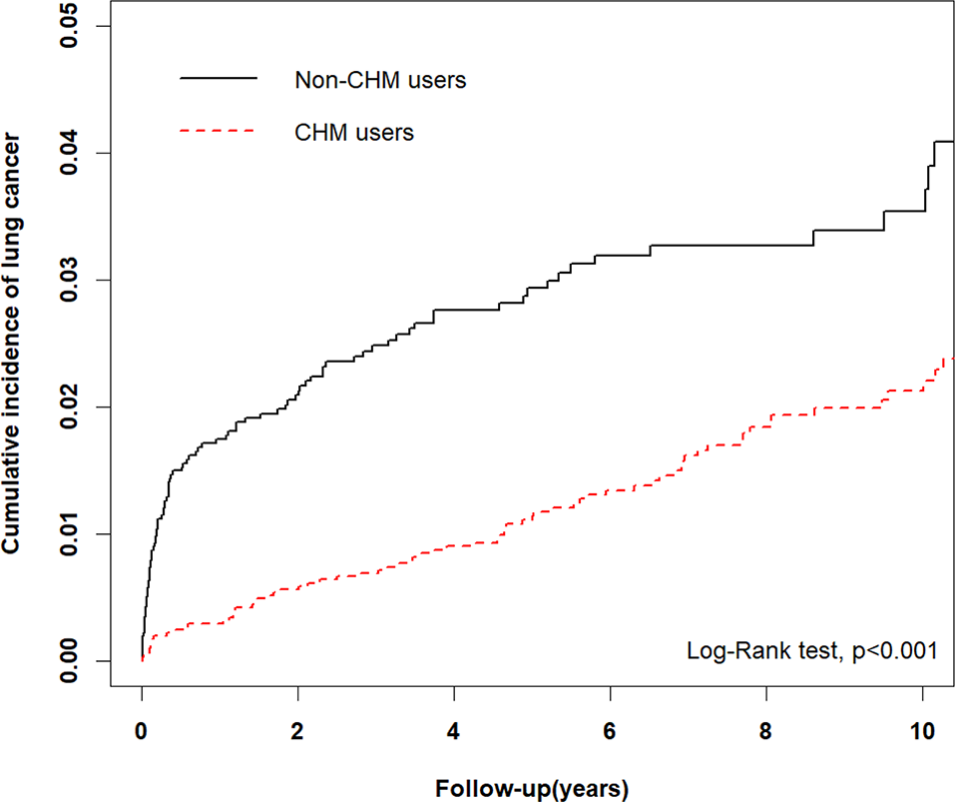
Comparison of Kaplan-Meier estimates of the 10-year cumulative incidence of lung cancer in COPD patients with and without CHM usage (Log-rank test, p < 0.001).

A total of 46 lung cancer events occurred among CHM users and 64 among non-CHM users (**Table 2**). COPD patients using CHM treatment had a significantly lower risk for lung cancer compared with non-CHM users (adjusted hazard ratio [aHR] = 0.36, 95% CI = 0.24–0.53, p < 0.001). The risk of lung cancer was significantly higher for patients aged 40–59 years (aHR = 5.32, 95% CI = 2.19–12.94, p < 0.001) and those aged ≥60 years (aHR = 16.75, 95% CI = 7.54–37.23, p < 0.001) compared with patients aged 18–39 years (reference population). Type of occupation did not significantly affect the risk of developing lung cancer, whether COPD patients were receiving CHM treatment or not. We used Kaplan-Meier estimates to calculate cumulative incidence of lung cancer for the CHM and non-CHM groups over the 10-year follow-up period, adjusting for patients’ age, gender, type of occupation, and comorbidities. The cumulative incidence of lung cancer was significantly lower among CHM users than among non-CHM users (Log-rank test, p < 0.001) (**Figure 2**).

**Table 2:** Cox model measured hazard ratio and 95% confidence intervals of lung cancer associated with and without CHM and covariates among COPD patients.

Characteristics	Event no.	Crude			Adjusted		
	(n = 110)	HR	(95% CI)	p value	HR	(95% CI)	p value
CHM							
No	64	1	reference		1	reference	
Yes	46	0.47	(0.32–0.69)	<0.001	0.36	(0.24–0.53)	<0.001
Sex							
Female	43	1	reference		1	reference	
Male	67	1.22	(0.83–1.79)	0.30	1.32	(0.88–1.99)	0.18
Age at baseline							
18–39	7	1	reference		1	reference	
40–59	17	3.81	(1.58–9.20)	0.003	5.32	(2.19–12.94)	<0.001
≥60	86	10.50	(4.86–22.69)	<0.001	16.75	(7.54–37.23)	<0.001
Job type							
Office workers	42	1	reference		1	reference	
Manual workers	58	1.68	(1.13–2.50)	0.01	1.39	(0.93–2.08)	0.10
Others	10	1.23	(0.62–2.45)	0.56	0.93	(0.46–1.86)	0.83
Baseline comorbidity (Yes vs No)							
Hypertension	47	1.07	(0.73–1.56)	0.74	0.65	(0.43–0.99)	0.05
Stroke	4	0.64	(0.23–1.73)	0.37	0.50	(0.18–1.39)	0.19
Coronary artery disease	35	1.54	(1.03–2.31)	0.03	1.20	(0.78–1.85)	0.41
Diabetes mellitus	8	0.76	(0.37–1.57)	0.46	0.69	(0.33–1.44)	0.32
Hyperlipidemia	30	0.97	(0.64–1.48)	0.89	0.82	(0.53–1.28)	0.39
Chronic kidney disease	2	0.97	(0.24–3.93)	0.97	1.03	(0.25–4.23)	0.97
Osteoporosis	16	1.30	(0.77–2.22)	0.33	1.07	(0.6–1.88)	0.83
Depression	3	0.40	(0.13–1.27)	0.12	0.62	(0.18–2.09)	0.44
Anxiety	16	0.61	(0.36–1.04)	0.07	0.60	(0.34–1.06)	0.08
Dementia	1	0.88	(0.12–6.32)	0.90	0.71	(0.10–5.17)	0.73
Smoking related diseases	1	2.65	(0.37–19.04)	0.33	3.53	(0.48–25.75)	0.21

HR, hazard ratio; CI, confidence interval; CHM, Chinese herbal medicine.

Adjusted HR: adjusted for gender, age, type of occupation, and all comorbidities in Cox proportional hazards regression.

### The Top 10 Single Herbs and Formulas Used in CHM Prescriptions

**Figure 3:**
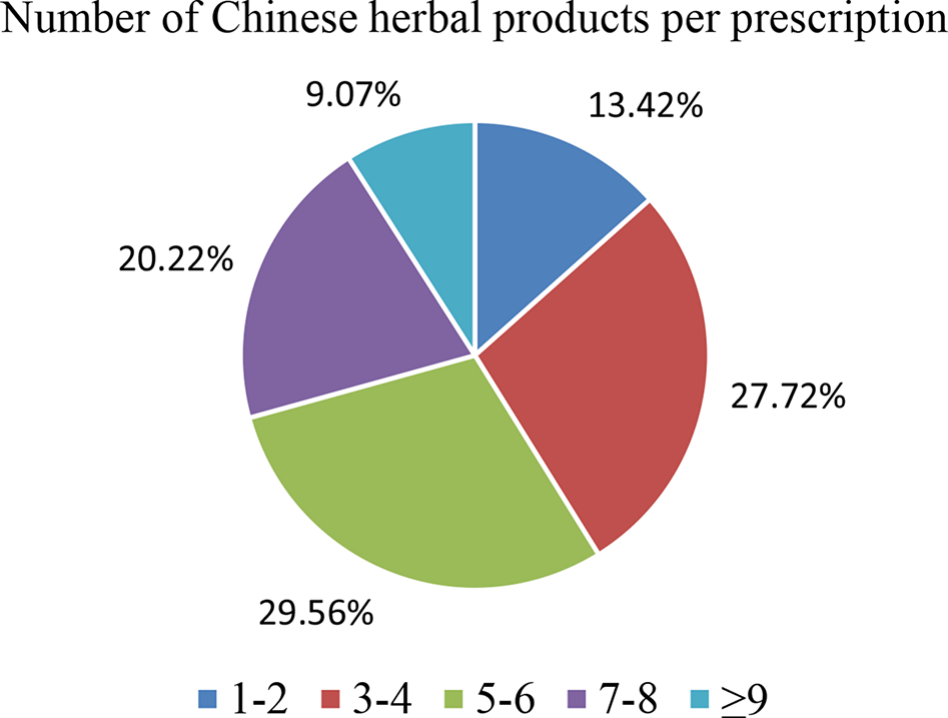
Distribution of Chinese herbal medicine combinations in one treatment for all patients with COPD.

**Figure 4:**
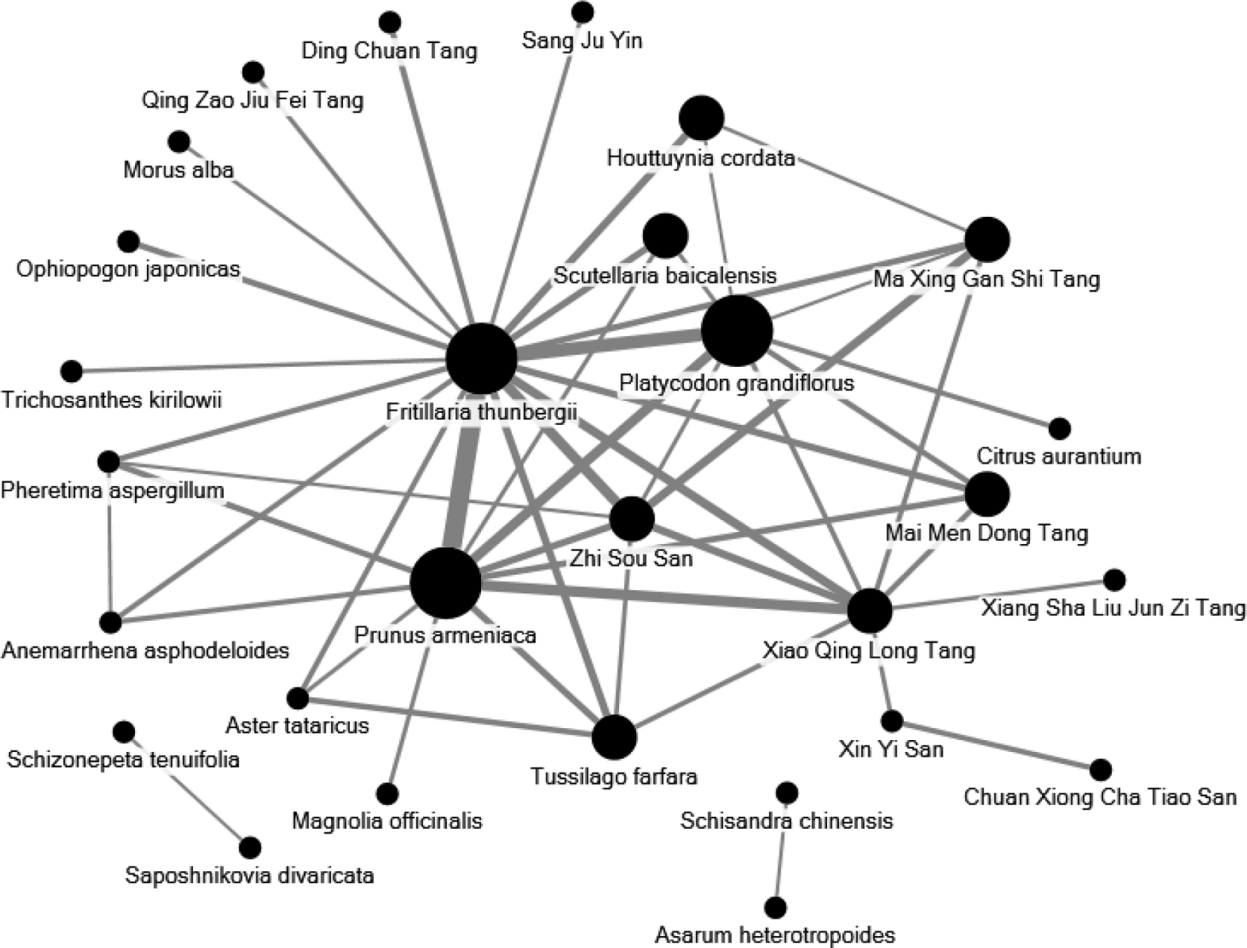
Network analyses of the most frequent 30 herbs and formulas combinations for all patients with COPD. The size of spot indicates the frequency of Chinese herbal product and the width of line indicates the combination time of two Chinese herbal products.

The 10 most commonly used single herbs in COPD patients were *Fritillaria thunbergii*, *Prunus armeniaca*, *Platycodon grandiflorus*, *Scutellaria baicalensis*, *Ophiopogon japonicus*, *Houttuynia cordata*, *Salvia miltiorrhiza*, *Glycyrrhiza uralensis*, *Tussilago farfara*, and *Magnolia officinalis* (**Table 3**). Xiao Qing Long Tang, Zhi Sou San, Mai Men Dong Tang, Ma Xing Gan Shi Tang, Ding Chuan Tang, Bai He Gu Jin Tang, Xin Yi Qing Fei Tang, Qing Zao Jiu Fei Tang, Qing Fei Tang, and Xin Yi San were the 10 most common formulas used for COPD patients (**Table 4**). Approximately 30% of prescriptions for COPD patients contained five or six CHMs (**Figure 3**). COPD patients most often received prescriptions containing three to eight CHMs. The most common combination of single herbs was *F. thunbergii* and *P. armeniaca*, followed by *F. thunbergii* and *P. grandiflorus*. Xiao Qing Long Tang was the most common formula to be combined with other CHMs (**Figure 4**).

**Table 3:** Ten most common herbs prescribed for patients with COPD.

Scientific name (Chinese name)	Frequency	Number of person-days	Average daily dose (g)	Average duration for prescription (days)
Fritillaria thunbergii (Zhe-Bei-Mu)	7,611	74,223	1.3	9.8
Prunus armeniaca (Xing-Ren)	5,902	58,205	1.2	9.9
Platycodon grandiflorus (Jie-Geng)	5,380	56,919	1.6	10.6
Scutellaria baicalensis (Huang-Qin)	3,146	31,050	1.2	9.9
Ophiopogon japonicus (Mai-Men-Dong)	2,641	28,296	2.9	10.7
Houttuynia cordata (Yu-Xing-Cao)	2,886	27,082	1.4	9.4
Salvia miltiorrhiza (Dan-Shen)	1,831	25,080	1.7	13.7
Glycyrrhiza uralensis (Gan-Cao)	2,063	21,753	1.4	10.5
Tussilago farfara (Kuan-Dong-Hua)	2,430	21,681	1.2	8.9
Magnolia officinalis (Hou-Po)	1,867	21,558	1.3	11.5

**Table 4:** Ten most common formulas prescribed for patients with COPD.

Formula name	Frequency	Number of person-days	Average daily dose (g)	Average duration for prescription (days)
Xiao Qing Long Tang	7,407	82,110	5	11.1
Zhi Sou San	6,180	51,301	4.6	8.3
Mai Men Dong Tang	4,750	44,566	5.1	9.4
Ma Xing Gan Shi Tang	5,085	41,541	6.5	8.2
Ding Chuan Tang	3,878	40,226	7.2	10.4
Bai He Gu Jin Tang	3,601	35,653	8.3	9.9
Xin Yi Qing Fei Tang	3,035	32,173	5.8	10.6
Qing Zao Jiu Fei Tang	3,048	30,848	5.7	10.1
Qing Fei Tang	2,602	25,936	5.9	10
Xin Yi San	2,268	25,623	6.8	11.3

### The Degree of CHM Exposure as Treatment Duration

We have analysed the degree of exposure as CHM treatment duration. In this study, all CHM users were those with more than 14 days of CHM prescriptions. The risk of lung cancer was associated with cumulative use (in days) of CHM. Compared to non-CHM users, CHM users who had between 28 and 60 days of CHM were at lower risk of developing lung cancer (aHR = 0.32, 95% CI = 0.18–0.57) (**Table 5**).

**Table 5:** Hazard Ratios and 95% confidence intervals of lung cancer risk associated with cumulative use day of CHM among COPD patients.

	Event no. (n = 110)	Person-year	IR	Hazard ratio (95% CI)	
				Crude	Adjusted^†^
**Non-CHM users**	64	12,527	51.09	Ref.	Ref.
**Chinese herb users**					
14-28 days	12	4,494	26.70	0.60(0.32–1.11)	0.53(0.28–1.02)
28-60 days	14	8,625	16.23	0.36(0.20–0.65)**	0.32(0.18–0.57)**
>60 days	20	8,602	23.25	0.52(0.31–0.86)*	0.41(0.25–0.69)**

Crude HR^＊^ represented relative hazard ratio; Adjusted HR^†^ represented adjusted hazard ratio: mutually adjusted for age, gender, job type and comorbidities in Cox proportional hazard regression.

*p < 0.05, **p < 0.01, ***p < 0.001.

CHM, Chinese herbal medicine; CI, confidence interval; IR, incidence rates.

### Risk of Incident Lung Cancer According to Use of Western Medicine and CHM

We compared the risk of incident lung cancer according to use/non-use of Western medicine and CHM (**Table 6**). In analyses adjusted for demographic and comorbidity factors, patients using only CHM were at significantly lower risk of developing lung cancer compared to patients using Western medications for COPD (long-acting beta agonists [LABAs], long-acting muscarinic antagonists [LAMAs], short-acting beta-agonists [SABAs], short-acting muscarinic antagonists [SAMAs] and inhaled corticosteroids [ICS]) and no CHM (p < 0.001 for all comparisons). Moreover, patients using ICS combined with CHM were also at lower risk of lung cancer (aHR = 0.43, 95% CI = 0.20–0.92). However, patients using SABAs without CHM treatment had a significantly higher risk of developing lung cancer (aHR = 2.25, 95% CI = 1.32–3.84).

**Table 6:** Cox proportional hazard regression analysis for the risk of lung cancer associated with COPD medication.

Variable		Event	Person years	IR	Crude HR (95% CI)	Adjusted HR (95% CI)
**CHM**	**LABA**					
No	No	59	11,840	49.83	Ref.	Ref.
No	Yes	5	687	72.77	1.55(0.62–3.88)	1.32(0.53–3.31)
Yes	No	38	19,787	19.20	0.44(0.30–0.66)***	0.38(0.25–0.58)***
Yes	Yes	8	1,935	41.35	0.96(0.46–2.01)	0.55(0.261.18)
**CHM**	**LAMA**					
No	No	60	12,285	48.84	Ref.	Ref.
No	Yes	4	242	165.11	3.64(1.32–10.03)*	2.48(0.89–6.93)
Yes	No	42	20,901	20.09	0.47(0.32–0.70)***	0.40(0.27–0.60)***
Yes	Yes	4	820	48.77	1.13(0.41–3.12)	0.57(0.20–1.61)
**CHM**	**SABA**					
No	No	42	11,043	38.03	Ref.	Ref.
No	Yes	22	1,484	148.25	3.38(2.01–5.67)***	2.25(1.32–3.84)**
Yes	No	26	18,478	14.07	0.41(0.25–0.67)***	0.35(0.21–0.58)***
Yes	Yes	20	3,243	61.67	1.80(1.05–3.07)*	1.06(0.60–1.85)
**CHM**	**SAMA**					
No	No	50	11,433	43.73	Ref.	Ref.
No	Yes	14	1,094	128.01	2.48(1.37–4.50)**	1.50(0.81–2.77)
Yes	No	28	19,252	14.54	0.37(0.23–0.59)***	0.32(0.20–0.51)***
Yes	Yes	18	2,470	72.88	1.84(1.07–3.17)*	0.98(0.56–1.73)
**CHM**	**ICS**					
No	No	59	11,578	50.96	Ref.	Ref.
No	Yes	5	949	52.68	1.09(0.44–2.72)	0.89(0.35–2.22)
Yes	No	38	19,143	19.85	0.45(0.30–0.67)***	0.39(0.25–0.59)***
Yes	Yes	8	2,579	31.02	0.70(0.33–1.46)	0.43(0.20–0.92)*

IR, incidence rates, per 10,000 person-years; HR, hazard ratio; CI, confidence interval; LABA, long-acting beta-agonists; LAMA, long-acting muscarinic antagonists; SABA, short-acting beta-agonists; SAMA, short-acting muscarinic antagonists; ICS, inhaled corticosteroids.

Adjusted HR: adjusted for gender, age, job type and all comorbidities in Cox proportional hazards regression.

*p value < 0.05; **p value < 0.01; ***p value < 0.001.

## Discussion

COPD is a persistently inflammatory response in the airways and lung parenchyma accompanied by excess mucus secretion, fibrosis, and proteolysis, leading to the clinical phenotypes of chronic bronchitis, airway obstruction, and emphysema (Mannino et al., 2006). COPD increases the risk of lung cancer (Takiguchi et al., 2014). In an investigation of cancer trends in COPD patients in primary care settings, the annual incidence rate of lung cancer was 4-fold to 5-fold higher in patients with COPD compared with the general population; moreover, the COPD cohort had worse 3-year survival from lung cancer than those in the general population (Kiri et al., 2009b). Smoking is the main risk factor for COPD and lung cancer (Brashier and Kodgule, 2012). Other risk factors include long-term exposure to air pollution, occupational dust, previous tuberculosis, early childhood recurrent lower respiratory infections, poor nutrition, and old age (Brashier and Kodgule, 2012). Quitting smoking is the best way to prevent COPD and lung cancer (Tønnesen, 2013; Underner et al., 2014). The number of global COPD cases continues to increase: a recent meta-regression analysis estimated a global COPD prevalence of 10.7% (7.3%–14.0%) in 1990 and 11.7% (8.4%–15.0%) in 2010, with 2.27 million and 3.84 million affected cases in 1990 and 2010, respectively (Adeloye et al., 2015).

Our study revealed significantly higher rates of hypertension (p = 0.03) among CHM users than among non-CHM users (**Table 1**). In Taiwan, TCM is commonly used to assist in the treatment of these diseases. Some studies have mentioned that arterial hypertension may be related to the development of renal cell carcinoma, but not lung cancer (Koene et al., 2016; Radisauskas et al., 2016). In our study, the mean age of patients in the CHM cohort was significantly older than those in the non-CHM cohort (57.7 years vs 55.9 years; p < 0.001). Statistical analyses have indicated that age is an independent risk factor for lung cancer, particularly age above 60 years (Saldias Penafiel et al., 2016; Sandelin et al., 2018). These findings are consistent with our study data; older age was positively correlated with the risk of lung cancer (**Table 2**). However, this pattern was reversed in the CHM group, who had a lower risk of lung cancer.

Modern medicine provides diverse treatment modalities for COPD. Bronchodilators alone or in combination with anti-inflammatory agents, such as corticosteroids, are the main therapeutic strategy. We compared the effects of Western medicine and CHM on the risk of developing lung cancer in COPD patients (**Table 6**). Some reports have suggested that regular use of ICS in COPD patients may reduce the risk of lung cancer (Parimon et al., 2007; Kiri et al., 2009a; Raymakers et al., 2019), which is consistent with our research. However, long-term exposure to steroids is associated with severe side effects including severe infection such as pneumonia or steroid-induced fractures (Loke et al., 2011; Suissa et al., 2013). Moreover, COPD patients with tuberculosis or pneumonia are at increased risk of lung cancer after using ICS (Wu et al., 2016a). COPD treatment guidelines emphasize the need to lower comorbidities and risk of exacerbations, as well as increase the quality of life and exercise capacity of patients (Putcha et al., 2015). Control of COPD disease without treatment-related side effects is essential and this has resulted in the concurrent use of CHM and conventional Western medical treatment in Taiwan. A LABA/LAMA combination can reduce COPD exacerbations and effectively improve symptoms and quality of life scores (Oba et al., 2018). It is also the best combination to improve FEV_1_ (Aziz et al., 2018). A population-based study has mentioned that the combination of a LAMA and a LABA may increase the risk of stroke compared with use of either agent alone (Tsai et al., 2015). These agents are deemed to be safe when used appropriately in COPD patients without uncontrolled cardiovascular disease or other notable comorbidities (Lahousse et al., 2016). However, as shown in this study (**Table 6**), the use of LABAs, LAMAs, SABAs or SAMAs as monotherapy were associated with an apparently higher risk of lung cancer. With the addition of CHM to these agents, the risk of lung cancer was reduced. This novel, important finding needs to be confirmed with further, more rigorous research.

In TCM theory, use of Chinese medicine is determined by “syndrome differentiation and treatment”. The three most commonly used CHM herbs are *F. thunbergii* (Zhe-Bei-Mu), *P. armeniaca* (Xing-Ren), and *P. grandiflorus* (Jie-Geng). *F. thunbergii* is used to expel heat and phlegm in TCM treatment; this herb has also shown antitussive, expectorant, and anti-inflammatory effects in murine models (Wang et al., 2012). *P. armeniaca* is used to relieve coughing and gasping for breath, to nourish the lungs, and to relieve constipation. *P. grandiflorus* facilitates flow of lung-*qi*, relieves sore throat, eliminates sputum, and expels pus from the lungs and throat. *P. grandiflorus* root-derived saponin inhibits acrolein-induced expression of mucin 5 and inhibits reactive oxygen species (ROS) formation (Choi et al., 2011). Flavones of *S. baicalensis* (Huang-Qin) have demonstrated an anti-cancer effect by arresting the cell cycle and suppressing the inflammatory environment and production of oxygen-derived radicals (Li-Weber, 2009). *O. japonicus* (Mai-Men-Dong) is used to nourish the yin and moisten the lung. Study evidence has revealed a wide range of pharmacologic activities associated with *O. japonicus*, including anti-oxidation, anti-inflammation, cardiovascular protection, cough relief, immunoregulatory, and antidiabetic properties, and antitumor activity (Chen et al., 2016). Ophiopogonin D’, a bioactive saponin compound isolated from *O. japonicus*, induces cell apoptosis by inhibiting the nuclear factor (NF)-κB pathway or suppressing the STAT3 signaling pathway and downregulating the expression of oncogenic genes (Lee et al., 2018a; Lee et al., 2018b). *H. cordata* (Yu-Xing-Cao) clears away heat, detoxifies, eliminates phlegm, and drains pus. In a mouse model of lipopolysaccharide (LPS)-induced acute lung injury (ALI), *H. cordata* and flavonoid constituents, including afzelin, hyperoside and quercitrin, inhibit lung inflammatory responses (Lee et al., 2015). In a cigarette smoke and LPS-induced COPD rat model, sodium houttuyfonate, an active compound extracted from *H. cordata Thunb*, is associated with marked anti-inflammatory effects related to suppression of the toll-like receptor 4 (TLR4)/NF-κB pathway (Wu et al., 2017). *S. miltiorrhiza *(Dan-Shen) is used to relieve pain, promote blood circulation, and nourish blood. Many studies have shown that *S. miltiorrhiza* or its extract has antioxidative, anti-inflammatory, and even antitumor activities (Hung et al., 2016; XD et al., 2019). Moreover, *S. miltiorrhiza* components such as Tan IIA can markedly reduce the area of collagen deposition in bleomycin-induced pulmonary fibrosis and inhibit TGF-β1-triggered alveolar EMT in rat models, indicating a positive effect for prevention and treatment of lung fibrotic change associated with COPD (He et al., 2015; Tang et al., 2015). Traditionally, *G. uralensis* (Gan-Cao) has been used to replenish *qi*, clear away heat, detoxify, relieve cough, and reconcile various medicines. The major antitussive and expectorant compounds extracted from *G. uralensis* are liquiritin apioside and liquiritin, which can significantly decrease cough frequency (Kuang et al., 2018). Glycyrrhizin not only decreases levels of pro-inflammatory cytokines such as interleukin (IL)-1β and TNF-α, but also suppresses the expression of cyclooxygenase-2 (COX-2) and iNOS, reducing the severity of LPS-induced ALI in mice (Ni et al., 2011), and improves long-term histopathologic changes in a murine model of asthma (Hocaoglu et al., 2011). *T. farfara* (Kuan-Dong-Hua) is one of the major ingredients of the TCM formulas Zhi Sou San and Ding Chuan Tang, which have proven significant antitussive, expectorant, and anti-inflammatory effects in recent pharmacological research (Wu et al., 2016b). Recent *in vitro* investigations have shown that tussilagone, a natural product derived from *T. farfara*, inhibits the production of mucin protein and downregulates the expression of the mucin gene in airway epithelial cells *via* the NF-κB signaling pathway (Choi et al., 2018). *M. officinalis* (Hou-Po) is used to regulate *qi* and eliminate flatulence. It is traditionally believed that intestinal gas also affects the function of lungs. Recent studies have revealed that the product magnolol, purified from *M. officinalis*, has antioxidant, anti-inflammatory, and antitumor effects, even inducing apoptosis in non-small cell lung cancer (NSCLC) cells (Tsai et al., 2014).

Xiao Qing Long Tang (XQLT), the most commonly used formula in our study, is capable of downregulating pro-inflammatory cytokines, iNOS expression and NF-kB phosphorylation in cigarette smoke concentrate-stimulated human airway epithelial cells (Shin et al., 2018). In another study, XQLT inhibited the growth of A549 cells in NSCLC by inducing cell apoptosis (Park et al., 2015). Zhi Sou San and Mai Men Dong Tang both act as antitussive agents. In the treatment of cough, compared to conventional cough medicines, Zhi Sou San was associated with fewer adverse effects and a lower recurrence rate (Cheng et al., 2017). Mai Men Dong Tang attenuates cough severity in chronic cough of COPD (Mukaida et al., 2011) and minimizes airway hyper-responsiveness by suppressing the release of vagal neuroeffector transmitters (Aizawa et al., 1999; Aizawa et al., 2003). Ma Xing Gan Shi Tang and Ding Chuan Tang have traditionally been used to treat cough or asthma in the pattern of “lung heat”. In acute exacerbations of COPD, Ding Chuan Tang combined with Western anti-infective agents can effectively control clinical symptoms, reduce the recurrence rate and improve lung function (Jing, 2011). In another clinical study examining acute exacerbations of COPD, Ma Xing Gan Shi Tang combined with conventional treatment lowered TNF-α and IL-6 concentrations and increase FEV_1_% and FEV_1_/FVC% (Huang et al., 2012). In a mouse model of H1N1 influenza A virus-associated ALI, Ma Xing Gan Shi Tang ameliorates lung cell apoptosis and downregulates serum TNF-α concentrations (Zhong et al., 2016). Bai He Gu Jin Tang is commonly used in the “lung and kidney yin deficiency” type of lung diseases, although there are few studies related to COPD. However, investigations using modified Bai He Gu Jin Tang to treat lung cancer have indicated increased efficacy and reduced toxicity of chemotherapy, with an improved quality of life (Hu et al., 2007; Liu et al., 2012). Xin Yi Qing Fei Tang and Xin Yi San have often been used in allergic rhinitis, acute and chronic rhinitis, sinusitis, and other such conditions, but few studies are related to COPD (Yen et al., 2015a; Yen et al., 2015b). Qing Zao Jiu Fei Tang is used to treat the lung disease of “dry evil injured lung syndrome”, and is good for coughing. In an evaluation of TCM combined with conventional cancer treatment for lung cancer, Qin Zao Jiu Fei Tang was the most effective TCM for mortality reduction (HR = 0.81; 95% CI = 0.72–0.91) (Liao et al., 2017). *In vitro* and *in vivo* studies have revealed that the molecular mechanisms underlying the antitumor activities of Qin Zao Jiu Fei decoction are associated with the upregulation of p53 mRNA and downregulation of c-myc and Bcl-2 mRNA expression, as well as suppression of matrix metallopeptidase 9 (MMP-9), vascular endothelial growth factor (VEGF), and VEGF receptor protein expression (Xie et al., 2018). In Japan, Qing Fei Tang (Seihai-to) has been found to increase airway ciliary beat frequency (Kogiso et al., 2018) and reduce oxygen radical production in inflamed lungs of rats (Iwasaki et al., 1999). All of these herbs and formulas have been used to alleviate clinical symptoms, decrease inflammation and/or prevent carcinogenesis in COPD patients, based on both TCM theory and scientific studies, thus, a summary of common formulas and herbs is given in **Supplementary Table 2**. When compared with conventional treatment alone in stable COPD, TCM combined with conventional treatment is associated with improvements in FEV_1_, the six-minute-walk, St George’s Respiratory Questionnaire (SGRQ) scores and fewer exacerbations (Haifeng et al., 2015). To our knowledge, chronic inflammation, including increased oxidative stress, persistent exposure to pro-inflammatory cytokines and an impaired DNA repair mechanism, is a leading cause of lung cancer, to the same extent as is seen with COPD progression. (Durham and Adcock, 2015).

Our study evidence suggests that the duration of CHM exposure in COPD is important: patients used CHM for more than 28 days had a significantly reduced risk of lung cancer compared with non-CHM users. This is worthy of further investigation in clinical observational studies.

Whether TCM treatment lowers the risk of lung cancer in patients with COPD is unclear. Our study evidence shows that CHM not only reduces COPD-related symptoms, but also has antitumor effects by relieving oxidative stress, reducing tumor cell apoptosis and lowering concentrations of pro-inflammatory cytokines. According to a previous study, tumor progression in NSCLC correlates with tumor-associated macrophages (TAMs), which are regulated by IL-10 and programmed death-ligand 1 (PD-L1) (Pang et al., 2017). In that study, CHM not only showed inhibitory effects upon TAMs in the tumor microenvironment, but also alleviated lung cancer-related symptoms (Pang et al., 2017).

There are some noteworthy limitations in this study. The NHIRD dataset does not contain details of body mass index values, levels of physical activity, environmental/chemical exposure, or family medical history. In addition, the NHIRD does not provide more detailed clinical evaluations including COPD assessment or pulmonary function tests, serum laboratory data, imaging data, pathological reports or cancer staging. Nevertheless, this large-scale, population-based, retrospective cohort study offers valuable insights into the effectiveness of CHM combined with Western medicine in the reduction of risk for lung cancer among patients with COPD.

## Conclusion

In conclusion, COPD and lung cancer could represent different symptoms and signs, with the same predisposition. Our findings suggest that CHM might retard the progression of COPD to lung cancer and that CHM could provide a new treatment option for patients with COPD. In combination with Western medicine for the treatment of COPD, CHM may reduce the incidence of lung cancer. Further studies are warranted to explore the possible antitumor effects and associated mechanisms of CHM treatment in patients with COPD.

## Data Availability

The raw data supporting the conclusions of this manuscript will be made available by the authors, without undue reservation, to any qualified researcher.

## Ethics Statement

This study was approved by the Review Board and Ethics Committee of China Medical University Hospital, Taichung, Taiwan (CMUH104-REC2-115(CR-2)). The review board waived the requirement to sign informed consent from patients as all of the data were de-identified.

## Author Contributions

T-HL and S-IC wrote the manuscript and reviewed the literature. Y-CS and M-CL collected, assembled and analyzed the data. H-JL interpreted the data and offered administrative support. S-TH designed and conceived the study and amended the manuscript. All of the authors approved the final manuscript.

## Funding

This work was supported and funded by Health and Welfare Surcharge of Tobacco Products, China Medical University Hospital Cancer Research Center of Excellence (MOHW108-TDU-B-212-124024), China Medical University Hospital (DMR-108-007, DMR-108-009, DMR-108-044, DMR-107-164 and CRS-106-001) and the Chinese Medicine Research Center, China Medical University under the Higher Education Sprout Project, Ministry of Education (CMRC-CHM-1) in Taiwan. This work was also supported by grants from the Ministry of Health and Welfare, Taiwan (MOHW107-TDU-B-212-123004), China Medical University Hospital, Academia Sinica Stroke Biosignature Project (BM10701010021), MOST Clinical Trial Consortium for Stroke (MOST 107-2321-B-039 -004-), Tseng-Lien Lin Foundation, Taichung, Taiwan, and Katsuzo and Kiyo Aoshima Memorial Funds, Japan.

## Conflict of Interest Statement

The authors declare that the research was conducted in the absence of any commercial or financial relationships that could be construed as a potential conflict of interest.

## Abbreviations

COPD, chronic obstructive pulmonary disease; CHM, Chinese herbal medicine; TCM, traditional Chinese medicine; FEV_1_, forced expiratory volume in one second; FVC, forced vital capacity; RNOS, reactive nitrogen and oxygen species; EMT, epithelial-mesenchymal transition; HIF-1α, hypoxia-inducible factor-1-alpha; NF-κB, nuclear factor kappa B; MAPK, mitogen-activated protein kinase; iNOS, inducible nitric oxide synthase; CHPs, Chinese herbal products; CRP, C-reactive protein; ALI, acute lung injury; XQLT, Xiao Qing Long Tang; TAMs, tumor-associated macrophages; TLR4, Toll-like receptor-4; STAT3, signal transducer and activator of transcription 3; IL-1β, interleukin 1 beta; IL-6, interleukin 6; IL-8, interleukin 8; IL-10, interleukin 10; TNF-α, tumor necrosis factor alpha; TGF-β1, transforming growth factor beta 1; LPS, lipopolysaccharides; MMP-9, matrix metallopeptidase 9; VEGF, vascular endothelial growth factor; NSCLC, non-small cell lung cancer; LABA, long-acting beta-agonist; LAMA, long-acting muscarinic antagonist; SABA, short-acting beta-agonist; SAMA, short-acting muscarinic antagonist.

## References

[B1] Aberle D. R. Adams A. M. Berg C. D. Black W. C. Clapp J. D. Fagerstrom R. M. (2011). Reduced lung-cancer mortality with low-dose computed tomographic screening. N. Engl. J. Med. 365 (5), 395–409. 10.1056/NEJMoa1102873 21714641 PMC4356534

[B2] Adcock I. M. Caramori G. Barnes P. J. (2011). Chronic obstructive pulmonary disease and lung cancer: new molecular insights. Respiration 81 (4), 265–284. 10.1159/000324601 21430413

[B3] Adeloye D. Chua S. Lee C. Basquill C. Papana A. Theodoratou E. (2015). Global and regional estimates of COPD prevalence: Systematic review and meta-analysis. J. Glob. Health 5 (2), 020415. 10.7189/jogh.05.020415 26755942 PMC4693508

[B4] Aizawa H. Shigyo M. Nakano H. Matsumoto K. Inoue H. Hara N. (1999). Effect of the Chinese herbal medicine, Bakumondo-to, on airway hyperresponsiveness induced by ozone exposure in guinea-pigs. Respirology 4 (4), 349–354. 10.1046/j.1440-1843.1999.00203.x 10612567

[B5] Aizawa H. Yoshida M. Inoue H. Hara N. (2003). Traditional oriental herbal medicine, Bakumondo-to, suppresses vagal neuro-effector transmission in guinea pig trachea. J. Asthma 40 (5), 497–503. 10.1081/JAS-120018779 14529099

[B6] An X. Zhang A. L. May B. H. Lin L. Xu Y. Xue C. C. (2012). Oral Chinese herbal medicine for improvement of quality of life in patients with stable chronic obstructive pulmonary disease: a systematic review. J. Altern. Complement Med. 18 (8), 731–743. 10.1089/acm.2011.0389 22803654 PMC3421964

[B7] Aziz M. I. A. Tan L. E. Wu D. B. Pearce F. Chua G. S. W. Lin L. (2018). Comparative efficacy of inhaled medications (ICS/LABA, LAMA, LAMA/LABA and SAMA) for COPD: a systematic review and network meta-analysis. Int. J. Chron. Obstruct Pulmon. Dis. 13, 3203–3231. 10.2147/COPD.S173472 30349228 PMC6186767

[B8] Bozinovski S. Vlahos R. Anthony D. McQualter J. Anderson G. Irving L. (2016). COPD and squamous cell lung cancer: aberrant inflammation and immunity is the common link. Br. J. Pharmacol. 173 (4), 635–648. 10.1111/bph.13198 26013585 PMC4742298

[B9] Brashier B. B. Kodgule R. (2012). Risk factors and pathophysiology of chronic obstructive pulmonary disease (COPD). J. Assoc. Physicians India 60 Suppl (Suppl), 17–21. 23155808

[B10] Bray F. Ferlay J. Soerjomataram I. Siegel R. L. Torre L. A. Jemal A. (2018). Global cancer statistics 2018: GLOBOCAN estimates of incidence and mortality worldwide for 36 cancers in 185 countries. CA Cancer J Clin 68 (6), 394–424. 10.3322/caac.21492 30207593

[B11] Chen M. H. Chen X. J. Wang M. Lin L. G. Wang Y. T. (2016). *Ophiopogon japonicus*— A phytochemical, ethnomedicinal and pharmacological review. J Ethnopharmacol 181, 193–213. 10.1016/j.jep.2016.01.037 26826325

[B12] Cheng N. Zhu J. Ding P. (2017). Clinical effects and safety of zhi sou san for cough: a meta-analysis of randomized trials. Evid. Based Complement. Alternat. Med. 2017, 9436352. 10.1155/2017/9436352 28798807 PMC5536149

[B13] Choi B. S. Kim Y. J. Yoon Y. P. Lee H. J. Lee C. J. (2018). Tussilagone suppressed the production and gene expression of MUC5AC mucin *via* regulating nuclear factor-kappa B signaling pathway in airway epithelial cells. Korean J. Physiol. Pharmacol. 22 (6), 671–677. 10.4196/kjpp.2018.22.6.671 30402027 PMC6205938

[B14] Choi J. H. Hwang Y. P. Han E. H. Kim H. G. Park B. H. Lee H. S. (2011). Inhibition of acrolein-stimulated MUC5AC expression by *Platycodon grandiflorum* root-derived saponin in A549 cells. Food Chem. Toxicol. 49 (9), 2157–2166. 10.1016/j.fct.2011.05.030 21664222

[B15] Durham A. Adcock I. (2015). The relationship between COPD and lung cancer. Lung Cancer 90 (2), 121–127. 10.1016/j.lungcan.2015.08.017 26363803 PMC4718929

[B16] Guan R. Wang J. Li Z. Ding M. Li D. Xu G. (2018). Sodium Tanshinone IIA sulfonate decreases cigarette smoke-induced inflammation and oxidative stress *via* blocking the activation of MAPK/HIF-1alpha signaling pathway. Front. Pharmacol. 9, 263. 10.3389/fphar.2018.00263 29765317 PMC5938387

[B17] Haifeng W. Hailong Z. Jiansheng L. Xueqing Y. Suyun L. Bin L. (2015). Effectiveness and safety of traditional Chinese medicine on stable chronic obstructive pulmonary disease: a systematic review and meta-analysis. Complement. Ther. Med. 23 (4), 603–611. 10.1016/j.ctim.2015.06.015 26275654

[B18] He H. Tang H. Gao L. Wu Y. Feng Z. Lin H. (2015). Tanshinone IIA attenuates bleomycin-induced pulmonary fibrosis in rats. Mol. Med. Rep. 11 (6), 4190–4196. 10.3892/mmr.2015.3333 25672255 PMC4394983

[B19] Hocaoglu A. B. Karaman O. Erge D. O. Erbil G. Yilmaz O. Bagriyanik A. (2011). Glycyrrhizin and long-term histopathologic changes in a murine model of asthma. Curr. Ther. Res. Clin. Exp. 72 (6), 250–261. 10.1016/j.curtheres.2011.11.002 24648593 PMC3957157

[B20] Hu D. Yang Q. Liu J. (2007). The clinical observation of additional baihe gujin fang for attenuation and synergy on topical late stage of lung cancer with chemotherapy [J]. J. N. C. Med. 39 (1), 81–82. 10.3969/j.issn.0256-7415.2007.01.056

[B21] Huang F. Wen X.-F. Shen Q. (2012). Efficacy observation of maxing shigan decoction in the treatment of chronic obstructive pulmonary disease in acute exacerbation stage. China Pharmacy 2012 (31), 33. 10.6039/j.issn.1001-0408.2012.31.29

[B22] Hung Y. C. Pan T. L. Hu W. L. (2016). Roles of reactive oxygen species in anticancer therapy with *salvia miltiorrhiza* bunge. Oxid Med. Cell. Longev 2016, 5293284. 10.1155/2016/5293284 27579153 PMC4989081

[B23] Iwasaki K. Wang Q. Satoh N. Yoshida S. Akaike T. Sekizawa K. (1999). Effects of qing fei tang (TJ-90) on aspiration pneumonia in mice. Phytomedicine 6 (2), 95–101. 10.1016/S0944-7113(99)80042-7 10374247

[B24] Jing L. (2011). Clinical observation of modified dingchuan decoction for acute exacerbation of chronic obstructive pulmonary disease [J]. J. N. C. Med. 43 (11), 22–24. 10.13457/j.cnki.jncm.2011.11.017

[B25] Kiri V. A. Fabbri L. M. Davis K. J. Soriano J. B. (2009a). Inhaled corticosteroids and risk of lung cancer among COPD patients who quit smoking. Respir. Med. 103 (1), 85–90. 10.1016/j.rmed.2008.07.024 18793832

[B26] Kiri V. A. Soriano J. B. Visick G. Fabbri L. M. (2009b). Recent trends in lung cancer and its association with COPD: an analysis using the UK GP research database. Prim. Care Respir. J. 19 (1), 57. 10.4104/pcrj.2009.00048 PMC6827612 19756330

[B27] Koene R. J. Prizment A. E. Blaes A. Konety S. H. (2016). Shared Risk Factors in Cardiovascular Disease and Cancer. Circulation 133 (11), 1104–1114. 10.1161/CIRCULATIONAHA.115.020406 26976915 PMC4800750

[B28] Kogiso H. Ikeuchi Y. Sumiya M. Hosogi S. Tanaka S. Shimamoto C. (2018). Seihai-to (TJ-90)-induced activation of airway ciliary beatings of mice: Ca(2+) modulation of cAMP-stimulated ciliary beatings *via* PDE1. Int. J. Mol. Sci. 19 (3), 658. 10.3390/ijms19030658 PMC5877519 29495403

[B29] Kuang Y. Li B. Fan J. Qiao X. Ye M. (2018). Antitussive and expectorant activities of licorice and its major compounds. Bioorg. Med. Chem. 26 (1), 278–284. 10.1016/j.bmc.2017.11.046 29224994

[B30] Lahousse L. Verhamme K. M. Stricker B. H. Brusselle G. G. (2016). Cardiac effects of current treatments of chronic obstructive pulmonary disease. Lancet Respir. Med. 4 (2), 149–164. 10.1016/S2213-2600(15)00518-4 26794033

[B31] Lee J. H. Ahn J. Kim J. W. Lee S. G. Kim H. P. (2015). Flavonoids from the aerial parts of *Houttuynia cordata* attenuate lung inflammation in mice. Arch. Pharm. Res. 38 (7), 1304–1311. 10.1007/s12272-015-0585-8 25743630

[B32] Lee J. H. Kim C. Lee S. G. Sethi G. Ahn K. S. (2018a). Ophiopogonin D, a Steroidal Glycoside Abrogates STAT3 Signaling Cascade and Exhibits Anti-Cancer Activity by Causing GSH/GSSG Imbalance in Lung Carcinoma. Cancers (Basel) 10 (11), 427. 10.3390/cancers10110427 PMC6265752 30413072

[B33] Lee J. H. Kim C. Lee S. G. Yang W. M. Um J. Y. Sethi G. (2018b). Ophiopogonin D, modulates multiple oncogenic signaling pathways, leading to suppression of proliferation and chemosensitization of human lung cancer cells. Phytomedicine 40, 165–175. 10.1016/j.phymed.2018.01.002 29496169

[B34] Li-Weber M. (2009). New therapeutic aspects of flavones: the anticancer properties of *Scutellaria* and its main active constituents Wogonin, Baicalein and Baicalin. Cancer Treat. Rev. 35 (1), 57–68. 10.1016/j.ctrv.2008.09.005 19004559

[B35] Liao Y.-H. Li C.-I. Lin C.-C. Lin J.-G. Chiang J.-H. Li T.-C. (2017). Traditional Chinese medicine as adjunctive therapy improves the long-term survival of lung cancer patients. J. Cancer Res. Clin. Oncol. 143 (12), 2425–2435. 10.1007/s00432-017-2491-6 28803328 PMC11819392

[B36] Liu L. Zhou Q. Kuang Y.-X. (2012). Effect of Baihegujin decoction on lung cancer patients’ quality of life (QOL). Sichuan Med. J. 33 (6), 959–961. 10.16252/j.cnki.issn1004-0501-2012.06.081

[B37] Loke Y. K. Cavallazzi R. Singh S. (2011). Risk of fractures with inhaled corticosteroids in COPD: systematic review and meta-analysis of randomised controlled trials and observational studies. Thorax 66 (8), 699–708. 10.1136/thx.2011.160028 21602540

[B38] Mannino D. M. Watt G. Hole D. Gillis C. Hart C. McConnachie A. (2006). The natural history of chronic obstructive pulmonary disease. Eur. Respir. J. 27 (3), 627–643. 10.1183/09031936.06.00024605 16507865

[B39] Masoud G. N. Li W. (2015). HIF-1α pathway: role, regulation and intervention for cancer therapy. Acta Pharmaceutica Sinica B 5 (5), 378–389. 10.1016/j.apsb.2015.05.007 26579469 PMC4629436

[B40] Miao Q. Cong X. Du Y. Wang B. Qiao C. (2016). Anti-inflammatory effects of Chinese herbal medicine on COPD: a systematic review. Lung Dis. Treat. 2 (107), 2472–1018.1000107. 10.4172/2472-1018.1000107

[B41] Mukaida K. Hattori N. Kondo K. Morita N. Murakami I. Haruta Y. (2011). A pilot study of the multiherb Kampo medicine bakumondoto for cough in patients with chronic obstructive pulmonary disease. Phytomedicine 18 (8–9), 625–629. 10.1016/j.phymed.2010.11.006 21177084

[B42] Ni Y. F. Kuai J. K. Lu Z. F. Yang G. D. Fu H. Y. Wang J. A. (2011). Glycyrrhizin treatment is associated with attenuation of lipopolysaccharide-induced acute lung injury by inhibiting cyclooxygenase-2 and inducible nitric oxide synthase expression. J. Surg. Res. 165 (1), E29–E35. 10.1016/j.jss.2010.10.004 21074783

[B43] Oba Y. Keeney E. Ghatehorde N. Dias S. (2018). Dual combination therapy versus long-acting bronchodilators alone for chronic obstructive pulmonary disease (COPD): a systematic review and network meta-analysis. Cochrane Database Syst. Rev. 12, Cd012620. 10.1002/14651858.CD012620.pub2 30521694 PMC6517098

[B44] Pang L. Han S. Jiao Y. Jiang S. He X. Li P. (2017). Bu Fei Decoction attenuates the tumor associated macrophage stimulated proliferation, migration, invasion and immunosuppression of non-small cell lung cancer, partially *via* IL-10 and PD-L1 regulation. Int. J. Oncol. 51 (1), 25–38. 10.3892/ijo.2017.4014 28534943 PMC5467788

[B45] Parimon T. Chien J. W. Bryson C. L. McDonell M. B. Udris E. M. Au D. H. (2007). Inhaled corticosteroids and risk of lung cancer among patients with chronic obstructive pulmonary disease. Am. J. Respir. Crit. Care Med. 175 (7), 712–719. 10.1164/rccm.200608-1125OC 17185647 PMC1899285

[B46] Park C. Hong S. H. Kim G. Y. Choi Y. H. (2015). So-Cheong-Ryong-Tang induces apoptosis through activation of the intrinsic and extrinsic apoptosis pathways, and inhibition of the PI3K/Akt signaling pathway in non-small-cell lung cancer A549 cells. BMC Complement. Altern. Med. 15 (1), 113. 10.1186/s12906-015-0639-y 25889185 PMC4397677

[B47] Putcha N. Drummond M. B. Wise R. A. Hansel N. N. (2015). Comorbidities and Chronic Obstructive Pulmonary Disease: Prevalence, Influence on Outcomes, and Management. Semin. Respir. Crit Care Med. 36 (4), 575–591. 10.1055/s-0035-1556063 26238643 PMC5004772

[B48] Radisauskas R. Kuzmickiene I. Milinaviciene E. Everatt R. (2016). Hypertension, serum lipids and cancer risk: a review of epidemiological evidence. Medicina (Kaunas) 52 (2), 89–98. 10.1016/j.medici.2016.03.002 27170481

[B49] Raymakers A. J. N. Sadatsafavi M. Sin D. D. FitzGerald J. M. Marra C. A. Lynd L. D. (2019). Inhaled corticosteroids and the risk of lung cancer in chronic obstructive pulmonary disease (COPD): a population-based cohort study. Eur. Respir. J. 53 (6), 1801257. 10.1183/13993003.01257-2018 30956205

[B50] Saldias Penafiel F. Elola Aranguiz J. M. Uribe Monasterio J. Morales Soto A. Diaz Patino O. (2016). Risk factors for the development of lung cancer in a cohort of adult smokers. Rev. Med. Chil. 144 (11), 1382–1390. 10.4067/S0034-98872016001100003 28394954

[B51] Sandelin M. Mindus S. Thuresson M. Lisspers K. Stallberg B. Johansson G. (2018). Factors associated with lung cancer in COPD patients. Int. J. Chron. Obstruct Pulmon. Dis. 13, 1833–1839. 10.2147/COPD.S162484 29922050 PMC5995277

[B52] Shin N. R. Kim C. Seo C. S. Ko J. W. Cho Y. K. Kim J. C. (2018). So-Cheong-Ryoung-Tang attenuates pulmonary inflammation induced by cigarette smoke in bronchial epithelial cells and experimental mice. Front. Pharmacol. 9, 1064. 10.3389/fphar.2018.01064 30298007 PMC6160558

[B53] Shinozuka N. Tatsumi K. Nakamura A. Terada J. Kuriyama T. (2007). The traditional herbal medicine Hochuekkito improves systemic inflammation in patients with chronic obstructive pulmonary disease. J. Am. Geriatr. Soc. 55 (2), 313–314. 10.1111/j.1532-5415.2007.01057.x 17302677

[B54] Siegel R. Miller K. Jemal A. (2017). Cancer statistics, 2017. CA Cancer J Clin 67 (1), 7–30. 10.3322/caac.21387 28055103

[B55] Suissa S. Patenaude V. Lapi F. Ernst P. (2013). Inhaled corticosteroids in COPD and the risk of serious pneumonia. Thorax 68 (11), 1029–1036. 10.1136/thoraxjnl-2012-202872 24130228 PMC3812880

[B56] Tønnesen P. (2013). Smoking cessation and COPD. Eur. Respir. Rev. 22 (127), 37–43. 10.1183/09059180.00007212 23457163 PMC9487432

[B57] Takiguchi Y. Sekine I. Iwasawa S. Kurimoto R. Tatsumi K. (2014). Chronic obstructive pulmonary disease as a risk factor for lung cancer. World J. Clin. Oncol. 5 (4), 660–666. 10.5306/wjco.v5.i4.660 25300704 PMC4129530

[B58] Tang H. He H. Ji H. Gao L. Mao J. Liu J. (2015). Tanshinone IIA ameliorates bleomycin-induced pulmonary fibrosis and inhibits transforming growth factor-beta-beta-dependent epithelial to mesenchymal transition. J. Surg. Res. 197 (1), 167–175. 10.1016/j.jss.2015.02.062 25911951

[B59] Tao H. Luo W. Pei H. Zhu S. Zhang M. Chen B. (2014). Expression and significance of hypoxia-inducible factor-1α in patients with chronic obstructive pulmonary disease and smokers with normal lung function. Xi Bao Yu Fen Zi Mian Yi Xue Za Zhi 30 (8), 852–855. 25108440

[B60] Tsai J. R. Chong I. W. Chen Y. H. Hwang J. J. Yin W. H. Chen H. L. (2014). Magnolol induces apoptosis *via* caspase-independent pathways in non-small cell lung cancer cells. Arch. Pharm. Res. 37 (4), 548–557. 10.1007/s12272-013-0232-1 23943503

[B61] Tsai M. J. Chen C. Y. Huang Y. B. Chao H. C. Yang C. J. Lin P. J. (2015). Long-acting inhaled bronchodilator and risk of vascular events in patients with chronic obstructive pulmonary disease in Taiwan population. Medicine (Baltimore) 94 (51), e2306. 10.1097/MD.0000000000002306 26705214 PMC4697980

[B62] Underner M. Perriot J. Peiffer G. (2014). Smoking cessation in smokers with chronic obstructive pulmonary disease. Rev. Mal. Respir. 31 (10), 937–960. 10.1016/j.rmr.2014.07.001 25496790

[B63] Wang D. Wang S. Chen X. Xu X. Zhu J. Nie L. (2012). Antitussive, expectorant and anti-inflammatory activities of four alkaloids isolated from Bulbus of *Fritillaria wabuensis* . J Ethnopharmacol 139 (1), 189–193. 10.1016/j.jep.2011.10.036 22101082

[B64] Wu M.-F. Jian Z.-H. Huang J.-Y. Jan C.-F. Nfor O. N. Jhang K.-M. (2016a). Post-inhaled corticosteroid pulmonary tuberculosis and pneumonia increases lung cancer in patients with COPD. BMC Cancer 16 (1), 778. 10.1186/s12885-016-2838-4 27724847 PMC5057453

[B65] Wu Q.-Z. Zhao D.-X. Xiang J. Zhang M. Zhang C.-F. Xu X.-H. (2016b). Antitussive, expectorant, and anti-inflammatory activities of four caffeoylquinic acids isolated from *Tussilago farfara* . Pharm. Biol. 54 (7), 1117–1124. 10.3109/13880209.2015.1075048 26439905

[B66] Wu Z. Tan B. Zhang H. Guo Y. Tu Y. Qiu F. (2017). Effects of sodium houttuyfonate on pulmonary inflammation in COPD model rats. Inflammation 40 (6), 2109–2117. 10.1007/s10753-017-0650-1 28812176

[B67] XD M. E. Cao Y. F. Che Y. Y. Li J. Shang Z. P. Zhao W. J. (2019). Danshen: a phytochemical and pharmacological overview. Chin J. Nat. Med. 17 (1), 59–80. 10.1016/S1875-5364(19)30010-X 30704625

[B68] Xie B. Xie X. Rao B. Liu S. Liu H. (2018). Molecular mechanisms underlying the inhibitory effects of qingzaojiufei decoction on tumor growth in lewis lung carcinoma. Integr. Cancer Ther. 17 (2), 467–476. 10.1177/1534735417694953 28617188 PMC6041919

[B69] Yen H. R. Liang K. L. Huang T. P. Fan J. Y. Chang T. T. Sun M. F. (2015a). Characteristics of traditional Chinese medicine use for children with allergic rhinitis: a nationwide population-based study. Int. J. Pediatr. Otorhinolaryngol. 79 (4), 591–597. 10.1016/j.ijporl.2015.02.002 25704847

[B70] Yen H. R. Sun M. F. Lin C. L. Sung F. C. Wang C. C. Liang K. L. (2015b). Adjunctive traditional Chinese medicine therapy for patients with chronic rhinosinusitis: a population-based study. Int. Forum Allergy Rhinol. 5 (3), 240–246. 10.1002/alr.21446 25511322

[B71] Zhong Y. Zhou J. Liang N. Liu B. Lu R. He Y. (2016). Effect of Maxing Shigan Tang on H1N1 Influenza A Virus-Associated Acute Lung Injury in Mice. Intervirology 59 (5-6), 267–274. 10.1159/000458726 28468008

[B72] Zhou R. Luo F. Lei H. Zhang K. Liu J. He H. (2016). Liujunzi Tang, a famous traditional Chinese medicine, ameliorates cigarette smoke-induced mouse model of COPD. J Ethnopharmacol 193, 643–651. 10.1016/j.jep.2016.09.036 27660011

