# Supplementary material for: Conventional Western Treatment Combined With Chinese Herbal Medicine Alleviates the Progressive Risk of Lung Cancer in Patients With Chronic Obstructive Pulmonary Disease: A Nationwide Retrospective Cohort Study
Source: Front Pharmacol. 2019 Sep 13;10:987. doi: 10.3389/fphar.2019.00987 (PMC6753872; doi:10.3389/fphar.2019.00987)
Supplement: Supplementary file 1 [file Table_1.docx]

SUPPLEMENTARY TABLE 1**∣**Work/occupation details

| Office worker | Public officials and workers in central non-business institutions, central public officials, national junior colleges, private colleges, private primary and secondary school faculty and workers, government agencies, school public officials, local public officials, non-profit or private employees, public institutional staff and workers |
| --- | --- |
| Manual worker | Employed by certain employers, trainees of vocational training institutions, self-employed operators, specialized occupational and technical personnel, self-executing practitioners, professional trade union members, seafarers' general unions or captains' guilds, farmers, water conservancy members, fishermen |
| Others | Family dependents of a soldier, military expenses students, soldier, alternative service, low-income, veteran, family dependents of a veteran, monk, religious, and residents of social welfare institutions |
