# Supplementary material for: Conventional Western Treatment Combined With Chinese Herbal Medicine Alleviates the Progressive Risk of Lung Cancer in Patients With Chronic Obstructive Pulmonary Disease: A Nationwide Retrospective Cohort Study
Source: Front Pharmacol. 2019 Sep 13;10:987. doi: 10.3389/fphar.2019.00987 (PMC6753872; doi:10.3389/fphar.2019.00987)
Supplement: Supplementary file 2 [file Table_2.docx]

**SUPPLEMENTARY TABLE 2∣**Common Chinese formulas and herbs with possible mechanisms or effects that protect COPD patients from developing lung cancer

| TCM name | Main Components | Possible Mechanisms or Effects |
| --- | --- | --- |
| *Fritillaria thunbergii*  Zhe-Bei-Mu 浙貝母 | Peimine, Peiminine | Trigger G_0_/G_1_ phase arrest and increase apoptosis by enhancing caspase-3 expression and reducing microvessel density in tumor tissues of lung cancer models [1].  Suppress the inflammatory response by mediating the expression of proinflammatory cytokines, including IL-1β, IL-6, IL-8, TNF-α, NF-κB, and their mediators TGF-β1, MMP-9, and TIMP-1 in lung tissues of COPD rat models [2]. |
| *Prunus armeniaca*  Xing-Ren杏仁 | Amygdalin, Emulsin | Inhibit quorum sensing in *Pseudomonas aeruginosa* culture and thus combat pulmonary infection [3]. |
| *Platycodon grandiflorus*  Jie-Gen桔梗 | Polygalacic, Platycodigenin, Platycoside C, Prosapogenin | Activate NK cells and decrease metastasis by inhibiting the adhesion of lung tumor cells to the basement membrane [4].  Promote cancer cell autophagy and regulate intracellular signaling to reduce proliferation in A549 cells [5]. |
| *Scutellaria baicalensis*  Huang-Qin黃芩 | Baicalin, Baicalein | Regulate the PI3K/AKT/NF-κB pathway to decrease airway remodeling in inflamed COPD models [6].  Induce cell cycle arrest and decrease free radicals and inflammatory cytokine expression [7]. |
| *Ophiopogon japonicus*  Mai-Men-Dong 麥門冬 | Methylophiopogonanone, ophiopogonin, ophiopogonanone | Block NF-κB and STAT3 signaling pathways to trigger cell apoptosis [8] and suppress oncogenic gene expression [9]. |
| *Houttuynia cordata*  Yu-Xing-Cao 魚腥草 | decanoyl acetaldehyde, lauric aldehyde, afzelin, hyperin, quercitrin | Inhibit lung inflammatory response in a mouse model of LPS-induced acute lung injury [10].  Anti-inflammatory effects via the suppression of the TLR4/NF-κB pathway in a rat model of COPD induced by cigarette smoke and LPS [11]. |
| *Salvia miltiorrhiza*  Dan-Shen 丹參 | Tanshinone IIA | Reduce the area of collagen deposition in bleomycin-induced pulmonary fibrosis and inhibit TGF-β1-triggered alveolar EMT in rat models [12, 13]. |
| *Glycyrrhiza uralensis*  Gan-Cao 甘草 | liquiritin apioside, liquiritin | Decrease proinflammatory cytokines such as IL-1β and TNF-α as well as suppress the expression of COX-2 and iNOS to reduce ALI in a mouse model of LPS-induced ALI [14]. |
| *Tussilago farfara*  Kuan-Dong-Hua 款冬花 | Tussilagone, Faradiol, Armiliot | Inhibit the production of mucin protein and downregulate mucin gene expression in airway epithelial cells via the NF-κB signaling pathway [15]. |
| *Magnolia officinalis*  Hou-Po 厚朴 | Honokiol, Magnolol | Antitumor effects include inducing cell apoptosis, anti-inflammatory and anti-oxidative mechanisms in NSCLC cells [16]. |
| Xiao Qing Long Tang  小青龍湯 | *Ephedra sinica, Paeonia lactiflora, Cinnamomum cassia, Glycyrrhiza uralensis,*  *Pinellia ternata, Zingiber officinale, Asarum sieboldii, Schisandra chinensis* | Induction of apoptosis in NSCLC cells [17].  Inhibition of proinflammatory cytokines via the NF-kB pathway [18].  Decrease serum concentrations of proinflammatory cytokines such as IL-4, IL-8, and TNF-α [19]. |
| Zhi Sou San  止嗽散 | *Platycodon grandifloras, Schizonepeta tenuifolia, Aster tataricus, Stemona sessilifolia, Cynanchum stauntonii*  *Glycyrrhiza uralensis, Citrus reticulata* | In a rat model: inhibit lung inflammation, increase expression of aquaporins, and decrease Muc5AC expression in lung [20]. |
| Mai Men Dong Tang  麥門冬湯 | *Ophiopogon japonicus, Pinellia ternata, Panax ginseng, Glycyrrhiza uralensis, Oryza sativa, Ziziphus jujuba* | Attenuates airway hyper-responsiveness and has bronchodilatory effects by relaxing bronchial smooth muscle and increasing cAMP [21, 22]. |
| Ma Xing Gan Shi Tang  麻杏甘石湯 | *Ephedra sinica, Prunus armeniaca,  Glycyrrhiza uralensis, Aqueous calcium sulfate* | Decrease IL-4, IL-8, and TNF-α in rats with COPD [23] and regulate the oxidative/antioxidative balance in bronchial and alveolar epithelium of COPD rats through deceasing the expression of r-GCS and NF-kB [24].  Relieve the bronchial contraction induced by acetylcholine/histamine [25]. |
| Ding Chuan Tang  定喘湯 | *Ginkgo biloba, Ephedra sinica, Tussilago farfara, Morus alba, Pinellia ternata, Perilla frutescens, Prunus armeniaca, Scutellaria baicalensis, Glycyrrhiza uralensis* | Suppression of inflammation through regulating IRAK/NF-κB, IRAK/AP-1 and TBK1/IRF3 pathways in lipopolysaccharide-stimulated macrophages [26]. |
| Bai He Gu Jin Tang  百合固金湯 | *Rehmannia glutinosa (dried), Rehmannia glutinosa (processed), Ophiopogon japonicus, Lilium albanicum, Paeonia lactiflora, Angelica sinensis, Fritillaria thunbergii, Glycyrrhiza uralensis, Scrophularia ningpoensis, Platycodon grandiflorus* | Improve FEV_1_/FVC [27] and decrease levels of IL-2 and IL-6 in tuberculosis [28]. |
| Xin Yi Qing Fei Tang  辛夷清肺湯 | *Magnolia biondii, Scutellaria baicalensis, Gardenia jasminoides, Ophiopogon japonicus, Lilium albanicum, Aqueous calcium sulfate, Anemarrhena asphodeloides, Glycyrrhiza uralensis, Eriobotrya japonica, Cimicifuga heracleifolia* | Antibacterial effects against *Streptococcus pneumonia* [29]. |
| Qing Zao Jiu Fei Tang  清燥救肺湯 | *Morus alba (leaf), Aqueous calcium sulfate, Glycyrrhiza uralensis, Sesamum indicum, Asini Corii Collas, Panax ginseng, Ophiopogon japonicus, Prunus armeniaca, Eriobotrya japonica* | Promote INF-γ and AQP5 protein expression, and decrease TNF-α expression after *Mycoplasma pneumoniae* infection in mice [30]. |
| Qing Fei Tang  清肺湯 | *Glycyrrhiza uralensis, Scutellaria baicalensis, Platycodon grandiflorus,*  *Poria, Citrus reticulata, Angelica sinensis,*  *Fritillaria thunbergii, Morus alba, Asparagus cochinchinensis, Gardenia jasminoides, Prunus armeniaca,*  *Ophiopogon japonicus, Schisandra chinensis, Zingiber officinale, Ziziphus jujuba, Bambusa tuldoides* | Enhance the defense mechanism of the lower airway in pneumonia [31] through increasing ciliary beat frequency in mouse ciliary cells [32]. |
| Xin Yi San  辛夷散 | *Ligusticum striatum, Akebia quinata,*  *Cimicifuga foetida, Angelica dahurica,*  *Glycyrrhiza uralensis, Saposhnikovia divaricata, Magnolia biondii,*  *Asarum sieboldii, Ligusticum sinense, Camellia sinensis* | Suppress serum IgE levels and increase production of IL-10, soluble intercellular adhesion molecule-1 and IL-8 in allergic rhinitis [33]. |

1. Wang, D., et al., *Evaluation of antitumor property of extracts and steroidal alkaloids from the cultivated Bulbus Fritillariae ussuriensis and preliminary investigation of its mechanism of action.* BMC Complementary and Alternative Medicine, 2015. **15**(1).

2. Wang, D., et al., *The Isosteroid Alkaloid Imperialine from Bulbs of Fritillaria cirrhosa Mitigates Pulmonary Functional and Structural Impairment and Suppresses Inflammatory Response in a COPD-Like Rat Model.* Mediators Inflamm, 2016. **2016**: p. 4192483.

3. Koh, K.H. and F.Y. Tham, *Screening of traditional Chinese medicinal plants for quorum-sensing inhibitors activity.* J Microbiol Immunol Infect, 2011. **44**(2): p. 144-8.

4. Lee, K.J., et al., *Inhibition of tumor invasion and metastasis by aqueous extract of the radix of Platycodon grandiflorum.* Food Chem Toxicol, 2006. **44**(11): p. 1890-6.

5. Yim, N.H., et al., *A platycoside-rich fraction from the root of Platycodon grandiflorum enhances cell death in A549 human lung carcinoma cells via mainly AMPK/mTOR/AKT signal-mediated autophagy induction.* J Ethnopharmacol, 2016. **194**: p. 1060-1068.

6. Xu, F., et al., *Scutellaria baicalensis Attenuates Airway Remodeling via PI3K/Akt/NF-kappaB Pathway in Cigarette Smoke Mediated-COPD Rats Model.* Evid Based Complement Alternat Med, 2018. **2018**: p. 1281420.

7. Li-Weber, M., *New therapeutic aspects of flavones: the anticancer properties of Scutellaria and its main active constituents Wogonin, Baicalein and Baicalin.* Cancer Treat Rev, 2009. **35**(1): p. 57-68.

8. Lee, J.H., et al., *Ophiopogonin D modulates multiple oncogenic signaling pathways, leading to suppression of proliferation and chemosensitization of human lung cancer cells.* Phytomedicine, 2018. **40**: p. 165-175.

9. Lee, J.H., et al., *Ophiopogonin D, a Steroidal Glycoside Abrogates STAT3 Signaling Cascade and Exhibits Anti-Cancer Activity by Causing GSH/GSSG Imbalance in Lung Carcinoma.* Cancers (Basel), 2018. **10**(11).

10. Lee, J.H., et al., *Flavonoids from the aerial parts of Houttuynia cordata attenuate lung inflammation in mice.* Archives of Pharmacal Research, 2015. **38**(7): p. 1304-1311.

11. Wu, Z., et al., *Effects of Sodium Houttuyfonate on Pulmonary Inflammation in COPD Model Rats.* Inflammation, 2017. **40**(6): p. 2109-2117.

12. Tang, H., et al., *Tanshinone IIA ameliorates bleomycin-induced pulmonary fibrosis and inhibits transforming growth factor-beta-beta-dependent epithelial to mesenchymal transition.* J Surg Res, 2015. **197**(1): p. 167-75.

13. He, H., et al., *Tanshinone IIA attenuates bleomycin-induced pulmonary fibrosis in rats.* Molecular Medicine Reports, 2015. **11**(6): p. 4190-4196.

14. Ni, Y.-F., et al., *Glycyrrhizin Treatment Is Associated with Attenuation of Lipopolysaccharide-Induced Acute Lung Injury by Inhibiting Cyclooxygenase-2 and Inducible Nitric Oxide Synthase Expression.* Journal of Surgical Research, 2011. **165**(1): p. e29-e35.

15. Choi, B.S., et al., *Tussilagone suppressed the production and gene expression of MUC5AC mucin via regulating nuclear factor-kappa B signaling pathway in airway epithelial cells.* Korean J Physiol Pharmacol, 2018. **22**(6): p. 671-677.

16. Tang, H., et al., *Discovery and synthesis of novel magnolol derivatives with potent anticancer activity in non-small cell lung cancer.* Eur J Med Chem, 2018. **156**: p. 190-205.

17. Cheol Park, S.H.H., Gi-Young Kim, Yung Hyun Choi, *So-Cheong-Ryong-Tang induces apoptosis through activation of the intrinsic and extrinsic apoptosis pathways, and inhibition of the PI3K/Akt signaling pathway in non-small-cell lung cancer A549 cells.* BMC Complementary and Alternative Medicine, 2015. **15**.

18. Shin, N.R., et al., *So-Cheong-Ryoung-Tang Attenuates Pulmonary Inflammation Induced by Cigarette Smoke in Bronchial Epithelial Cells and Experimental Mice.* Front Pharmacol, 2018. **9**: p. 1064.

19. Wu, Y.W., T. Liu and S.P. Liu., *Impact of Xiaoqinglong decoction on lung function and serum cytokines in patients with acute exacerbation of chronic obstructive pulmonary disease.* Med. Inno. China., 2015. **12**: p. 109-111.

20. Zhen, G., et al., *Effects of Modified Zhisou Powder on Airway Mucus Production in Chronic Obstructive Pulmonary Disease Model Rats with Cold-Dryness Syndrome.* Evid Based Complement Alternat Med, 2018. **2018**: p. 7297141.

21. Aizawa H, S.M., Nakano H, Matsumoto K, Inoue H, Hara N., *Effect of the Chinese herbal medicine, Bakumondo-to, on airway hyperresponsiveness induced by ozone exposure in guinea-pigs.* Respirology, 1999. **4**(4): p. 349-354.

22. Aizawa H, Y.M., Inoue H, Hara N., *Traditional oriental herbal medicine, Bakumondo-to, suppresses vagal neuro-effector transmission in guinea pig trachea.* Journal of Asthma, 2003. **40**(5): p. 497-503.

23. Zhang, W., X.Y. Zhang and Y.M. Shao., *Changes in the level of cytokine in rats with chronic obstructive pulmonary disease of phlegm heat cumber lung type after treatment of Maxing Shigan decoction. .* Chin. J. Clin. Rehab., 2006. **10**: p. 167-170.

24. Zhang W, Z.X., Shao YM., *Effects of TCM treatment according to syndrome differentiation on expressions of nuclear factor-kappaB and gamma-glutamylcysteine synthetase in rats with chronic obstructive pulmonary disease of various syndrome types.* Zhongguo Zhong Xi Yi Jie He Za Zhi.27(5):426-30., 2007. **27**: p. 426-30.

25. Lin, Y.C., C.W. Chang, and C.R. Wu, *Antitussive, anti-pyretic and toxicological evaluation of Ma-Xing-Gan-Shi-Tang in rodents.* BMC Complement Altern Med, 2016. **16**(1): p. 456.

26. Zhang, Y., et al., *Dingchuan tang essential oil inhibits the production of inflammatory mediators via suppressing the IRAK/NF-kappaB, IRAK/AP-1, and TBK1/IRF3 pathways in lipopolysaccharide-stimulated RAW264.7 cells.* Drug Des Devel Ther, 2018. **12**: p. 2731-2748.

27. Jian-gang, L.Y.-p.J., *Clinical Research on Baihe Gujin Decoction for Treating 50 Cases in Stabilization Period of Chronic Obstructive Pulmonary Disease.* Chinese Journal of Experimental Traditional Medical Formulae, 2013. **19**.

28. Siqing, S.X.L., *Baihe Gujin decoction combined with anti-tuberculosis on levels of IL-2、IL-6 and TNF-αin patients with pulmonary tuberculosis.* Shaanxi Journal of Traditional Chinese Medicine 2018. **39**.

29. Toru Konishi, M.M., Zhixia Jiang, Tetsuya Arai, Toshiaki Makino, *Antibacterial activity of Shin’iseihaito (Xin Yi Qing Fei Tang) against Streptococcus pneumoniae.* Pharmacognosy Journal, 2016. **8**(1).

30. Hui, W.Z.M.N.Y.Z.Y.L.W.X.Y., *Effect of Qingzao Jiufei decoction and its decomposing agent on lung inflammation-related factors in mice infected with Mycoplasma pneumoniae.* ACTA LABORATORIUM ANIMALIS SCIENTIA SINICA, 2018. **26**.

31. N. Mantani, Y.K., T. Kamata, N. Sekiya, Y. Shimada, K. Usuda, I. Sakakibara, N. Hattori, K. Terasawa, *Effect of Seihai-to, a Kampo medicine, in relapsing aspiration pneumonia – an open-label pilot study.* Phytomedicine, 2002. **9**.

32. Haruka Kogiso, Y.I., Masako Sumiya, Shigekuni Hosogi, Saori Tanaka, Chikao Shimamoto, Toshio Inui, Yoshinori Marunaka and Takashi Nakahari, *Seihai-to (TJ-90)-Induced Activation of Airway Ciliary Beatings of Mice: Ca2+ Modulation of cAMP-Stimulated Ciliary Beatings via PDE1.* International Journal of Molecular Sciences, 2018. **19**.

33. Yang, S.H., et al., *Traditional Chinese medicine, Xin-yi-san, reduces nasal symptoms of patients with perennial allergic rhinitis by its diverse immunomodulatory effects.* Int Immunopharmacol, 2010. **10**(8): p. 951-8.
